# Fiber Surface Modification Technology for Fiber-Optic Localized Surface Plasmon Resonance Biosensors

**DOI:** 10.3390/s120302729

**Published:** 2012-02-29

**Authors:** Qiang Zhang, Chenyang Xue, Yanling Yuan, Junyang Lee, Dong Sun, Jijun Xiong

**Affiliations:** 1 National Key Laboratory for Electronic Measurement Technology, North University of China, No.3 Xueyuan Road, Taiyuan 030051, Shanxi, China; E-Mails: zhangq0902@163.com (Q.Z.); xuechenyang@nuc.edu.cn (C.X.); yuanyanling239@163.com (Y.Y.); lijunyang401@163.com (J.L.); 2 Department of Mechanical and Biomedical Engineering, City University of Hong Kong, 83 Tat Chee Avenue Kowloon, Hong Kong, China

**Keywords:** fiber optic biosensors, localized surface plasmon resonance, star-shaped gold nanoparticles, self-assembly

## Abstract

Considerable studies have been performed on the development of optical fiber sensors modified by gold nanoparticles based on the localized surface plasmon resonance (LSPR) technique. The current paper presents a new approach in fiber surface modification technology for biosensors. Star-shaped gold nanoparticles obtained through the seed-mediated solution growth method were found to self-assemble on the surface of tapered optical fibers via amino- and mercapto-silane coupling agents. Transmitted power spectra of 3-aminopropyltrimethoxy silane (APTMS)-modified fiber were obtained, which can verify that the silane coupling agent surface modification method is successful. Transmission spectra are characterized in different concentrations of ethanol and gentian violet solutions to validate the sensitivity of the modified fiber. Assembly using star-shaped gold nanoparticles and amino/mercapto silane coupling agent are analyzed and compared. The transmission spectra of the gold nanoparticles show that the nanoparticles are sensitive to the dielectric properties of the surrounding medium. After the fibers are treated in *t*-dodecylmercaptan to obtain their transmission spectra, APTMS-modified fiber becomes less sensitive to different media, except that modified by 3-mercaptopropyltrimethoxy silane (MPTMS). Experimental results of the transmission spectra show that the surface modified by the gold nanoparticles using MPTMS is firmer compared to that obtained using APTMS.

## 1. Introduction

Highly efficient microsensing systems are in demand because of their lower cost and smaller risk in launch [1]. As useful tools for measurement and analysis, optical biosensors find wide application in biorobotics, healthcare, pharmaceuticals, environmental monitoring, homeland security, and battlefield use [2]. The use of biosensors for the diagnosis of diseases, food testing, and environmental detection of biological agents has increased remarkably over the past decades. A fiber optic localized surface plasmon resonance (FO-LPR) chemical and biochemical sensing platform has undergone rapid development recently [3,4]. To apply fiber optic technology to biosensors, numerous challenging issues must be addressed, including surface immobilization (e.g., analyte capture efficiency and elimination of nonspecific binding, among others) and detection format (e.g., direct binding, sandwich-type binding, and competitive binding) [5]. The current paper discusses the application of surface immobilization technology in fiber optical sensing. Surface plasmon resonance (SPR) technology is used to detect the concentration of biomolecules in solution, under the condition that surface immobilization on the tapered fiber is stable and uniform.

Great efforts have been devoted to the development of optical fiber biosensors for the determination of various analytes, such as DNA, proteins, antigens, and cells. These sensors are based on absorbance, reflectance, fluorescence, chemiluminescence, bioluminescence, or refractive index (RI) measurements [6]. Fiber optical sensing is a microanalysis technique that offers a number of important benefits, such as low dissipation of transmittance, resistance to interference from electromagnetism, and small size [7]. A considerable number of studies have been performed for the development of optical fiber sensors based on LSPR modified by gold nanoparticles on the fiber surface [8–11]. Light of a certain wavelength can be absorbed as soon as the sensitive unit touches the medium, which can then be used to analyze changes in the transmission spectra for obtaining information on the concentrations and quantities of solutions [12,13]. Because LSPR is sensitive to the changes in strength of the local electric field in different media, surface modification using metal nanoparticles plays an increasingly important role to improve sensitivity in the development of new sensing technology [10,14].

Star-shaped gold nanoparticles exhibit seemingly magical optical properties because of their specific patterns. Thus far, few applications of these star-shaped gold nanoparticles have been reported. Both 3-aminopropyltrimethoxy silane (APTMS) and 3-mercaptopropyltrimethoxy silane (MPTMS) are convenient to use as linkers in surface modification; thus, comparing the two agents is important. In the current paper, the seed-mediated solution growth method is utilized to obtain star-shaped gold nanoparticles that self-assemble on the surface of tapered optical fibers using amino- and mercapto-silane coupling agents. According to the theory that an electric field can enhance LSPR of the gold nanoparticles, the modified tapered fiber can be characterized through transmission spectroscopy in various media. If the modified fibers are sensitive to different medium components and densities, they can also be used to measure the density of the biomolecules.

References [15,16] showed that gold nanoparticles can self-assemble uniformly on a silicon wafer surface using amino- and mercapto-silane coupling agents. Gold nanoparticles were modified on wafer, which made their characterization using scanning electron microscopy (SEM), atomic force microscopy, and X-ray photoelectron spectroscopy more convenient. The SEM image of the gold nanoparticle-modified wafer in APTMS and the image of the wafer treated in *t*-dodecylmercaptan were compared. The comparison results show that, after treatment in *t*-dodecylmercaptan, no electrostatic force between the gold nanoparticle and amino group existed. SEM images of the MPMTS-modified wafer were also obtained for comparison. Results show that the *t*-dodecylmercaptan can wrap the whole Au-S groups.

The predominant optical characteristics of the gold nanoparticles can be utilized to improve the sensitivity of optical biosensors via surface modification. In the current paper, two modification methods for the tapered fibers, APTMS and MPTMS, are analyzed. The mechanism of the modifications on the fiber surface can be characterized through the transmission spectrum measurement of the fibers placed in different solutions. The unique contributions of the current work lie in the use of the star-shaped gold nanoparticles and the comparison of the two silane coupling agents. With the proposed surface modification methodology, we can achieve better coupling effects for the optical biosensors such as optical ring resonator biosensors and biosensors based on liquid-core ring resonators. The excellent optical performances of the gold nanoparticles can increase the sensitivity of these biosensors.

## 2. Principle

**Figure 1.**
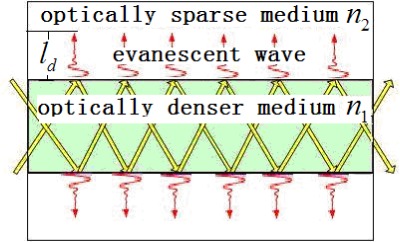
Penetration depth. The evanescent field decays in value of 1/e on the fiber surface.

LSPR is an important feature of metal nanoparticles, and can be generated by the evanescent field. Evanescent field strength and surface modification are the two central elements of the current research. LSPR of the fiber surface can be enhanced through properly choosing the light wavelength and the media of the evanescent field. Figure 1 illustrates light transmission in the fiber. The evanescent wave transmits exponentially in a vertical direction. The principle of the sensitivity improvement is that LSPR is sensitive to the change in strength of the local electric field in different media. Subsequently, the intensity of light when transmitted through the nanoparticle-modified fiber changes markedly in different media.

**Figure 2.**
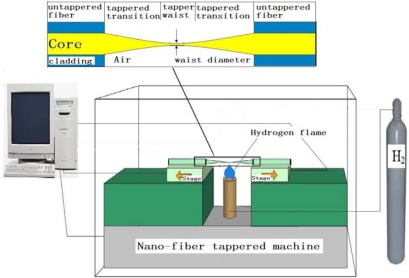
Schematic of fiber tapering.

Cladding of a single mode protects the light transmitting in the fiber. The cladding must be peeled off so that the evanescent field can interact with the surrounding environment. Meanwhile, the evanescent field on the surface of the naked fiber is too weak to enhance the LSPR of the gold nanoparticles. Tapering the fiber can be a good solution to enhance the evanescent wave. To taper the fiber, the two sides of the fiber are pulled while heating the naked fiber, as shown in Figure 2. A hydrogen flame is used to heat the naked fiber to its softening temperature. The pulling length and the constant amount of hydrogen are controlled using a computer program.

LSPR of the metal nanoparticles is closely related to the shape and size of the nanoparticles, as well as to the changes in the dielectric environment surrounding them. The star-shaped gold nanoparticles can increase the robustness of LSPR. In the current study, gold nanoparticles with a unique star-shaped structure are chosen to modify the tapered fibers.

**Figure 3.**
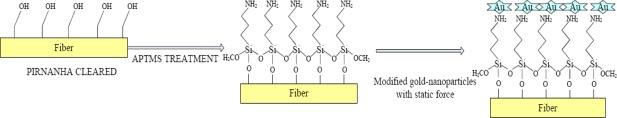
Schematic of self-assembly of gold nanoparticles in APTMS.

Gold nanoparticles can be modified by APTMS under electrostatic force, which is a type of intermolecular force. Polar molecules have dipole matrices and the electrostatic; interactive forces among the dipole molecules comprise the electrostatic force. Figure 3 illustrates the principle of surface modification using gold nanoparticles and self-assembly on the tapered fibers in APTMS. A piranha solution can remove the retained plastic cladding and transform the groups to hydroxyl on the fiber surface. After the fiber is treated with APTMS, amino groups are formed on the fiber surface [17]. The gold nanoparticles have negative electric charge, whereas the amino groups have positive electric charge. The produced electrostatic force between them results in the self-assembly of the gold nanoparticles on the treated fiber surface [18].

**Figure 4.**
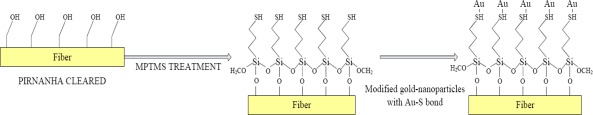
Schematic of gold nanoparticle self-assembly in MPTMS.

Figure 4 illustrates the principle of surface modification with gold nanoparticles and self-assembly procedures on the tapered fibers in MPTMS. MPTMS has methoxyl groups, whose hydrolysis product is silanol. Methoxyls interact with silanols absorbed on the surface. After the surface is washed to clear out the excess MPTMS, Si-O-Si bands form at high temperature, and self-assembled film with terminal mercapto groups is generated on the surface. Through the self-assembly process, gold nanoparticles can be modified using mercapto groups in Au-S bond [16].

Light loss during transmission spectrum measurement affects the strength of LSPR. The stronger the LSPR, the more light energy is absorbed by the molecules in the medium. In addition, LSPR is also affected by the surface modification conditions. Therefore, the transmission spectrum reflects the performance of the surface modification. The more uniformly and tightly tapered the fiber surfaces modified by the gold nanoparticles, the more sensitive the transmission spectra in different media are.

## 3. Methodology for Implementation

### 3.1. Reagents

MPTMS, *t*-dodecylmercaptan, Na3-citrate, ethanol, gentian violet, and sulfuric acid were purchased from Sinopharm Chemical Reagent Co., Ltd. (Shanghai, China). APTMS was obtained from Sigma. H_2_O_2_ was purchased from Chemical Reagent. Deionized water of approximately 18 MΩ·cm used in the current work was prepared using an ultrapure water machine UPR purchased from Ultra-pure Water Visible Ltd.

### 3.2. Apparatus

Single-mode optical fiber (SMF28e) was purchased from Corning. A multifunctional magnetic stirrer (MPL-CJ-88) was used to stir the solution. A constant temperature water bath (MPL-HWS) was used to maintain the gold sol at a certain temperature. A high-speed desktop centrifuge (TGL-16K) was used to centrifuge the gold sol to remove unreacted reagents. The transmission spectrum circuit consisting of a bromine tungsten light source (BFC-445), monochromator (SBP500), side window detector photomultiplier (PMTH-S1), and data acquisition system (DCS103) was purchased from Zolix Instruments Co., Ltd.

### 3.3. Surface Modification on Tapered Fibers

The tapered length of the single-mode optical fiber was 20 mm. The tapered fibers were first placed into piranha solution (30% H_2_O_2_ was added slowly to 98% concentrated sulfuric acid, with a volume ratio of 1:4) as surface treatment for 10 min at 80 °C. One of the optical fibers was placed in the acetic acid solution of MPTMS (1% by volume) for 2 h, whereas the other was placed in the acetic acid solution of APTMS (1% by volume) for surface silanization.

Under surface modification using APTMS and MPTMS, the star-shaped gold nanoparticles 80 nm to 120 nm in size were treated using the seed-mediated growth method [19]. The static material on the surfaces of the particles is the key of the self-assembly process, according to the binding mechanism.

The gold nanoparticles were divided into two portions. The portion for self-assembly on the MPTMS modified fiber was ultrasonically treated to remove static material on the surfaces of the particles. According to the mercapto group and particle linkage principle, the surfaces of the gold nanoparticles should be cleared first. Centrifugation must be performed to remove unreacted reagents. The surfaces of the silanized fibers were immersed in the treated gold sol for 12 h at room temperature. Tapered fiber modified by the gold nanoparticles with MPTMS was finally obtained. The portion for self-assembly on the APTMS modified fiber did not undergo ultrasonic treatment; centrifugation was performed directly. The rest of the steps were the same as that in MPTMS. Finally, optical fiber sensors functionalized by gold nanoparticles in APTMS were obtained.

**Figure 5.**
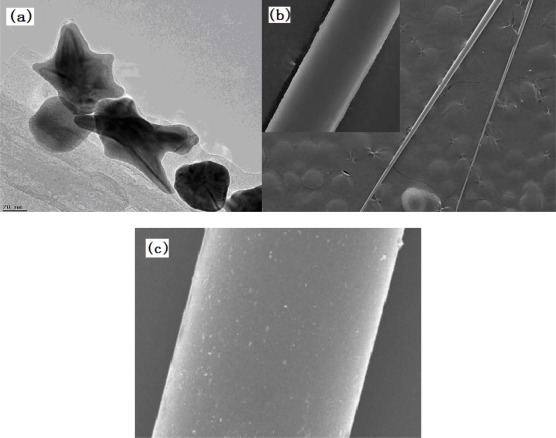
(**a**) TEM image of the star-shaped gold nanoparticles; (**b**) SEM image of the naked tapered fiber; and (**c**) SEM image of the tapered fiber modified with the nanoparticles.

Figure 5(a) shows the obtained star-shaped gold-nanoparticles 80 nm to 120 nm in size characterized using a transmission electron microscope. After conforming to the desired shape and size of the particles, the conditions of the surface modification are characterized via SEM, as shown in Figure 5(b,c).

### 3.4. Measurement of Transmission Spectra

The transmission spectra of the tapered and un-tapered fibers were tested. Theoretically, the transmission spectra peak value of the un-tapered fiber should be higher than that of the tapered fiber. This is because that the structure of the tapered fiber results in more light-leakage.

The transmitted power spectra of the silane coupling agent modified fiber were tested in air and water. Water absorption peaks appeared on the transmitted power spectra if the silane coupling agent absorbed the water molecules on the fiber surface. The absorption peaks of water indicate that the silane coupling agent was uniformly modified on the fiber. Because the modification methods of APTMS and MPTMS on the fibers surface were the same, only the transmitted power spectra of APTMS modified fiber were reported in the current paper.

**Figure 6.**
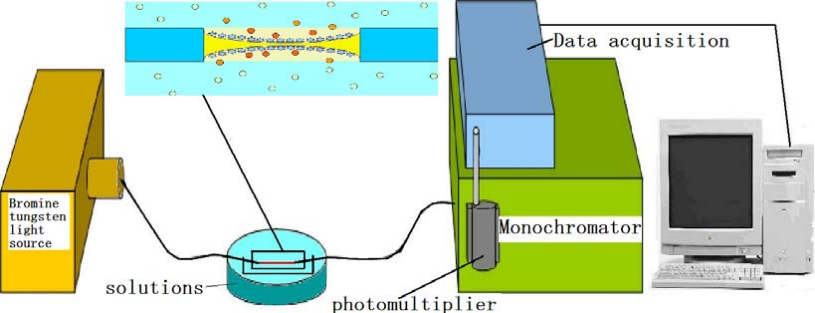
Diagram of the LSPR transmission spectrum measurement circuit.

The transmission spectra of the modified fibers vary in different media, depending on different RIs and dielectric conditions of the media. According to the transmission measurement principle, different modifications lead to different spectral distributions. Figure 6 presents a diagram of the circuit used for measuring the modified fibers in the transmission spectrum measurement. Due to the volatility of ethanol, different concentrations of gentian violet were tested. The ethanol concentration was 98%, and the gentian violet concentrations were 0.1 and 1 mM. To verify the two proposed modification methods, two modified fibers were treated in *t*-dodecylmercaptan for 24 h after the spectra were obtained. The transmission spectra of the modified fibers were then tested in the mentioned solutions. The characteristics of the transmission spectra were affected by several factors. The fiber connects the light source and monochromator via a fiber adapter. An extra part of the fiber must be cut such that the two ends of the fiber fit in the adapter. The cut of the fiber will lead to the change of the peak value of spectra. In the current work, the transmission spectra of the fibers modified by APTMS and MPTMS were tested and compared using the same fiber connection.

## 4. Results and Discussion

**Figure 7.**
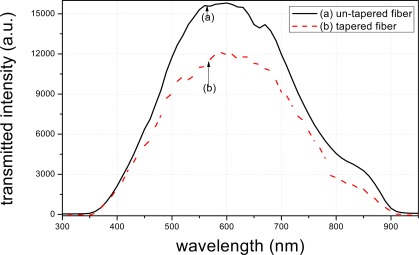
transmittance of tapered fiber and un-tapered fiber.

Figure 7 illustrates the transmission spectra of the tapered and un-tapered fibers. It is seen that the peak value of the transmitted intensity of the un-tapered fiber is more than 15,000 a.u., which is higher than that of the tapered fiber. This result indicates that the structure of the tapered fiber results in more light-leakage, and meanwhile, the evanescent field is enhanced through fiber tapering.

**Figure 8.**
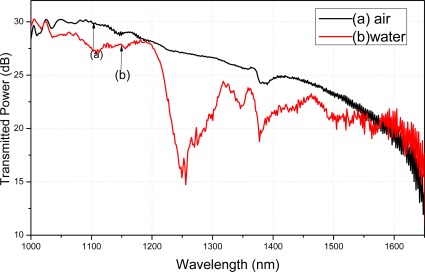
Transmitted power spectra of the silane coupling agent modified fiber tested in air and water.

Figure 8 illustrates the transmitted power spectra of a single-mode tapered fiber tested in air and water, modified with APTMS as a function of wavelength. The obvious decline in water *λ* = 1,240 nm and *λ* = 1,380 nm are pronounced, and are shown as the curve (b). The absorption peaks of water are approximately 1,240 and 1,380 nm [20]. Comparing the curve (b) with the curve (a), approximately 14.6 dB and 10.1 dB fall at *λ* = 1,240 nm and *λ* = 1,380 nm. The obvious decline in water illustrates that the APTMS on the fiber surface can absorb the water molecules. Depending on the successful APTMS surface modification, the gold nanoparticles can be absorbed by the silane coupling agent on the fiber surface.

The spectra of the fiber modified using APTMS after gold-nanoparticle conjugation are shown in Figure 8. Curve (a) denotes the spectrum of the modified fiber in air. Curve (b) represents the result when the modified fiber was placed in 98% ethanol; a clear decrease compared to curve (a) is evident. Curve (c) shows the result when the modified fiber was placed in 0.1 mM gentian violet. Curve (d) shows the result when the concentration was raised to 1 mM. Different concentrations resulted in different peak values in the transmission spectra. Furthermore, the transmission spectra of the fibers modified by the gold nanoparticles in APTMS varied when the solutions were changed.

**Figure 9.**
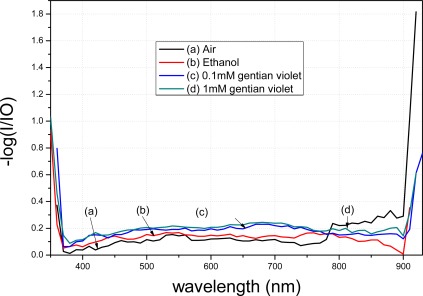
Transmittance of the modified fiber by APTMS.

**Figure 10.**
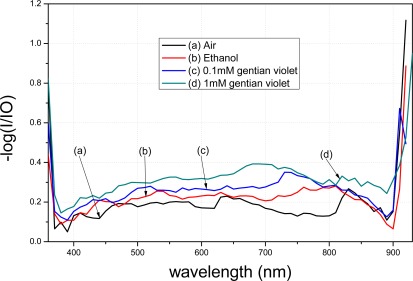
Transmittance of the modified fiber by MPTMS.

A comparison between Figures 9 and 10 shows that the transmission spectra of the modified tapered fiber using MPTMS decrease more obviously than when using APTMS. In order to better analyze the data, the y-axis is presented as −log (I/I0). I denotes the intensity of light exiting the fiber, where the gold nanoparticle-modified fiber is immersed in a medium. I_0_ denotes the intensity of light exiting a bare fiber, where there is no gold nanoparticle on the core surface and the fiber is immersed in a blank. The absorption peak of gentain violet is approximately 590 nm [21].

With the same reagent but with different concentrations, the MPTMS-modified fiber decreased by 0.048 a.u. at 590 nm, whereas the APTMS-modified fiber decreased by merely 0.015 a.u. at 590 nm. Based on the measurement principle of the transmission spectra that the distribution of the spectra represents the characteristics of the surface modification, these results indicate that surface modification of the gold nanoparticles using MPTMS is better than that using APTMS.

**Figure 11.**
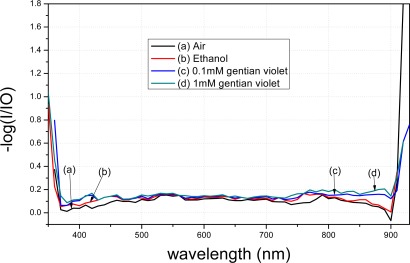
Transmittance of the modified fiber by APTMS after treatment with *t*-dodecylmercaptan.

Figure 11 illustrates the transmission spectra of the modified fiber treated with *t*-dodecylmercaptan. Gold nanoparticles modified using APTMS were further tested for comparison; results are shown in Figure 9. The gold nanoparticles were wrapped with *t*-dodecylmercaptan to remove electrostatic force. Figure 11 shows that the spectra decrease slightly under the same conditions of Figure 9 because LSPR of the gold nanoparticles continued to be absent from the fiber surface. This result shows that an electrostatic force exists between the nanoparticles and amino groups.

**Figure 12.**
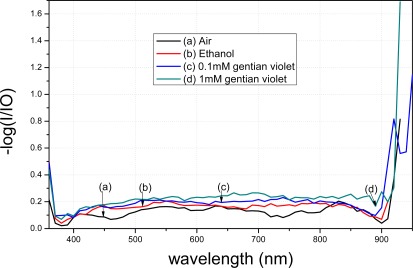
Transmittance of the modified fiber by MPTMS treated with t-dodecyl mercaptan.

After the MPTMS-modified nanoparticle fiber was treated with *t*-dodecylmercaptan, the spectra of the treated fiber markedly varied in different solutions, as shown in Figure 12.

Notably, the force between the gold-nanoparticles and mercapto group was not electrostatic. Based on a study of MPTMS-modified nanoparticles in Au-S on wafer [16], MPTMS is able to modify nanoparticles in Au-S on the fiber surface.

**Figure 13.**
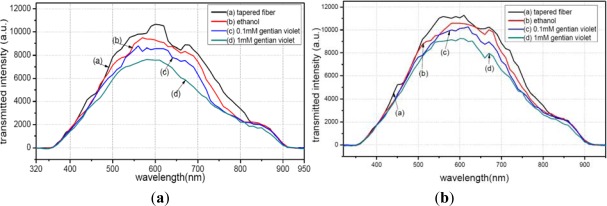
Transmittance of the modified fiber by MPTMS in two tests. (**a**) 1st test (**b**) 2nd test.

To verify reproducibility, we further performed tests on another MPTMS-modified fiber. It is seen from Figure 13 that the two tests displayed similar results. The peak value of the transmission spectra changed greatly when the MPTMS-modified fibers were immersed in different media in both tests. Note that different conditions of the surface modification lead to different curve shapes. The peak values of the transmission spectra in Figure 13 are different because the tapering conditions of the two fibers are not the same. With the same reagent but with different concentrations, the MPTMS-modified fibers decreased by 900 a.u. at 590 nm wavelength for in both the tests.

**Figure 14.**
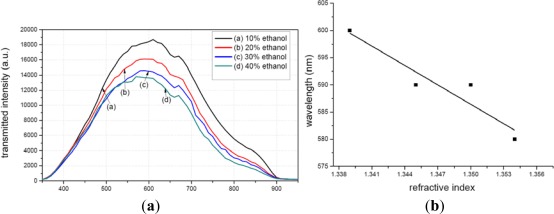
Refractive index sensitivity test. (**a**) Transmittance of the modified fiber by MPTMS; (**b**) Relationship between wavelength and refractive index.

Figure 14(a) illustrates the transmission spectra of the MPTMS-modified fiber tested in different concentrations of ethanol. The peak value occurs at different wavelengths for difference cases, *i.e.*, at 600 nm for 10% ethanol, 590 nm for 20% and 30% ethanol, and 580 nm for 40% ethanol. The refractive index is also different with different concentrations ethanol. Figure 14(b) illustrates the relationship between wavelengths and refractive index, based on which the refractive index sensitivity of MPTMS-modified fiber could be obtained as 1,190.5 nm/RIU.

## 5. Conclusions

This paper has presented the transmission spectra of the fibers modified using APTMS and MPTMS. Star-shaped gold nanoparticles 80 nm to 120 nm in size were modified on the surfaces of the tapered fibers using these reagents. Sensitivity of the resulting transmission spectra was tested. Results show that the transmission spectra of the tapered fiber modified using MPTMS decreased more than those modified using APTMS, which indicates that the surface modification of the gold nanoparticles using MPTMS is better than that using APTMS. This finding is consistent with that of gold nanoparticles modified by amino groups and mercapto groups in the Au-S bond. The reproducibility of this study and the refractive index sensitivity of MPTMS-modified fiber were also investigated. The proposed surface modification technology has great potential in the development and application of biosensors. The fibers become more sensitive to the different concentrations of molecules after surface modification because the predominant optical characteristics of the gold nanoparticles can enhance LSPR. As a result, the proposed surface modification technology can benefit the development of new optical biosensors with higher sensitivity. In future work, we will test biomolecules in different concentrations based on this modification method, and use the modified fiber to measure specific materials and concentrations.
